# Radiation-inducible miR-770-5p sensitizes tumors to radiation through direct targeting of PDZ-binding kinase

**DOI:** 10.1038/cddis.2017.116

**Published:** 2017-03-23

**Authors:** Hyung Chul Lee, Nam-Gu Her, Donghee Kang, Seung Hee Jung, Jinwook Shin, Minyoung Lee, In Hwa Bae, Young-Nyun Kim, Heon Joo Park, Young-Gyu Ko, Jae-Seon Lee

**Affiliations:** 1 Department of Molecular Medicine, Inha University College of Medicine, Incheon, Korea; 2 Hypoxia-related Disease Research Center, Inha University College of Medicine, Incheon, Korea; 3 Division of Life Sciences, Korea University, Seoul, Korea; 4 Department of Microbiology, Inha University College of Medicine, Incheon, Korea; 5 Division of Radiation Effects, Korea Institute of Radiological and Medical Sciences, Seoul, Korea; 6 Division of Basic Radiation Bioscience, Korea Institute of Radiological and Medical Sciences, Seoul, Korea; 7 Division of Cancer Biology, Research Institute, National Cancer Center, Goyang, Korea

## Abstract

Radiotherapy represents the most effective non-surgical modality in cancer treatment. MicroRNAs (miRNAs) are small non-coding RNAs that regulate gene expression, and are involved in many biological processes and diseases. To identify miRNAs that influence the radiation response, we performed miRNA array analysis using MCF7 cells at 2, 8, and 24 h post irradiation. We demonstrated that miR-770-5p is a novel radiation-inducible miRNA. When miR-770-5p was overexpressed, relative cell number was reduced due to increased apoptosis in MCF7 and A549 cells. Transcriptomic and bioinformatic analyses revealed that PDZ-binding kinase (PBK) might be a possible target of miR-770-5p for regulation of radiosensitivity. PBK regulation mediated by direct targeting of miR-770-5p was demonstrated using luciferase reporter assays along with wild-type and mutant PBK-3′untranslated region constructs. Radiation sensitivity increased and decreased in miR-770-5p- and anti-miR-770-5p-transfected cells, respectively. Consistent with this result, transfection of short interfering RNA against PBK inhibited cell proliferation, while ectopic expression of PBK restored cell survival from miR-770-5p-induced cell death. In addition, miR-770-5p suppressed tumor growth, and miR-770-5p and PBK levels were inversely correlated in xenograft model mice. Altogether, these data demonstrated that miR-770-5p might be a useful therapeutic target miRNA that sensitizes tumors to radiation via negative regulation of PBK.

Radiation therapy is one of the major treatment regimens for cancer patients. Approximately 50% of all cancer patients receive radiotherapy either alone or in combination with other treatment modalities such as surgery or chemotherapy.^1^ Ionizing radiation (IR) primarily induces damage to many cellular components, including DNA, protein, lipid, and other macromolecules by either direct or indirect generation of reactive oxygen species.^2^ Radiation-induced DNA damage initiates the DNA damage response (DDR), resulting in activation of multiple signaling checkpoint molecules such as ataxia telangiectasia mutated (ATM), ataxia telangiectasia and Rad3-related (ATR), checkpoint kinase 1 (CHK1), CHK2, and p53. The DDR coordinates repair and cell cycle progression, resulting in determination of cell fate between death and survival.^3, 4, 5^ Tumor suppressor p53, which is a key regulator in the DDR after IR exposure, undergoes post-translational modification to transcriptionally activate target genes such as Puma, Noxa, Gadd45, and p21.^6, 7, 8^ However, dysregulation of DDR and other self-repair mechanisms confer resistance to IR, thereby affecting the final outcome of radiation therapy in various cancers.^2, 9^ Therefore, recent strategies to improve the efficacy of radiation therapy have been actively developed by either antagonizing radiation-induced cellular defense mechanisms or reinforcing radiation-induced antiproliferative potential.

MicroRNAs (miRNAs) are small non-coding RNAs about ∼22 nucleotides in size that function as epigenetic controllers of cellular gene expression.^10^ The interaction between miRNAs and their target mRNAs through complementary base pairing exerts translational repression and mRNA degradation.^11, 12^ MiRNAs play important roles in various biological processes such as development, differentiation, cell proliferation, and cell death. Emerging roles of the miRNA signaling network in response to IR have been elucidated.^2, 9, 13^ For example, the p53-regulated miRNA miR-34a is one of the most important tumor-suppressing miRNA in cancer. MiR-34a sensitizes tumors to IR by targeting RAD51, a central regulator of DNA repair.^14^ The N-Myc-regulated miR-421 targets the 3′-untranslated region (3′UTR) of ATM mRNA and increases radiosensitivity.^15^ Elevation of miR-185 sensitizes cancer cells to radiation by targeting ATM and ATR.^16^ It was previously shown that miR-182 targets BRCA1 to impact homologous recombination-mediated DNA repair and increase cellular radiosensitivity.^17^ Currently, there is an increasing interest in defining functional miRNAs involving in the tumor radiation response to increase radiosensitivity.

In this study, we found that miR-770-5p is responsive to radiation in MCF7 breast carcinoma cells through miRNA microarray analysis. Specifically, miR-770-5p overexpression increases apoptosis via direct targeting of PDZ-binding kinase (PBK), and finally sensitizes radiation response both *in vitro* and *in vivo*. From our results, we propose that miR-770-5p as a potential miRNA for modulating the radiation response as well as a possible predictive biomarker.

## Results

### MiR-770-5p is an IR-inducible suppressor of cancer cell survival

**Figure 1:**
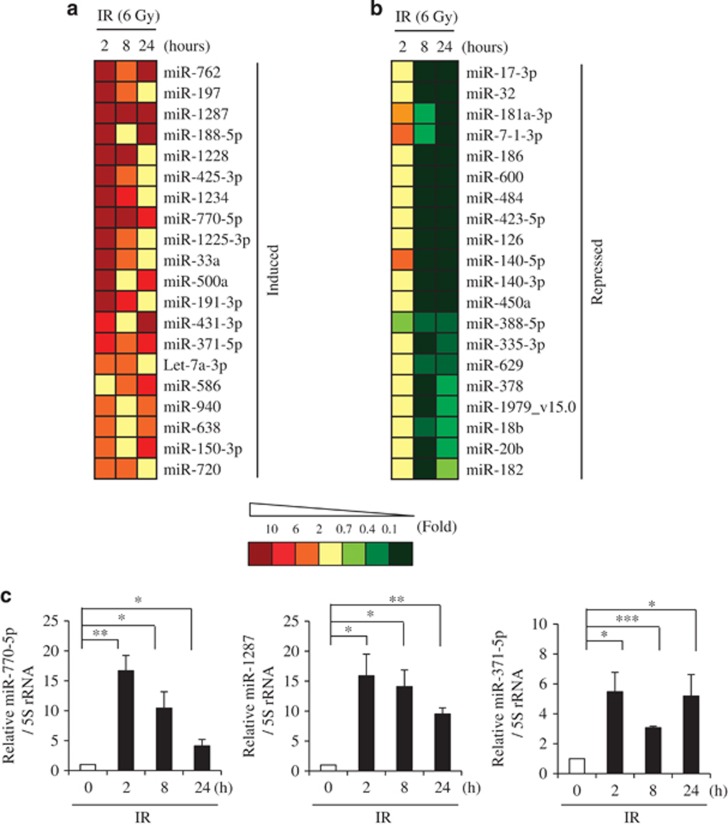
Upregulation of miR-770-5p upon IR exposure in breast cancer cells. (**a** and **b**) Heat map of IR-responsive miRNAs. MCF7 cells were collected at 0, 2, 8, and 24 h after 6 Gy of IR exposure, and miRNA array analysis was performed. Selected miRNAs, which were either induced or repressed by IR, are shown. Colored bars represent differential levels of miRNAs expressed in irradiated samples (2, 8, and 24 h) *versus* the non-irradiated sample (0 h). (**c**) Time-dependent induction of miR-770-5p, miR-1287, and miR-371-5p. MCF7 cells were exposed to 6 Gy of IR and collected at indicated time intervals (0, 2, 8, and 24 h). MiRNA levels were analyzed by qRT-PCR. 5S rRNA was used as internal control. Each bars represent means (*n* = 3) and S.D. (***P<0.001, **P<0.01, *P<0.05)

**Figure 2:**
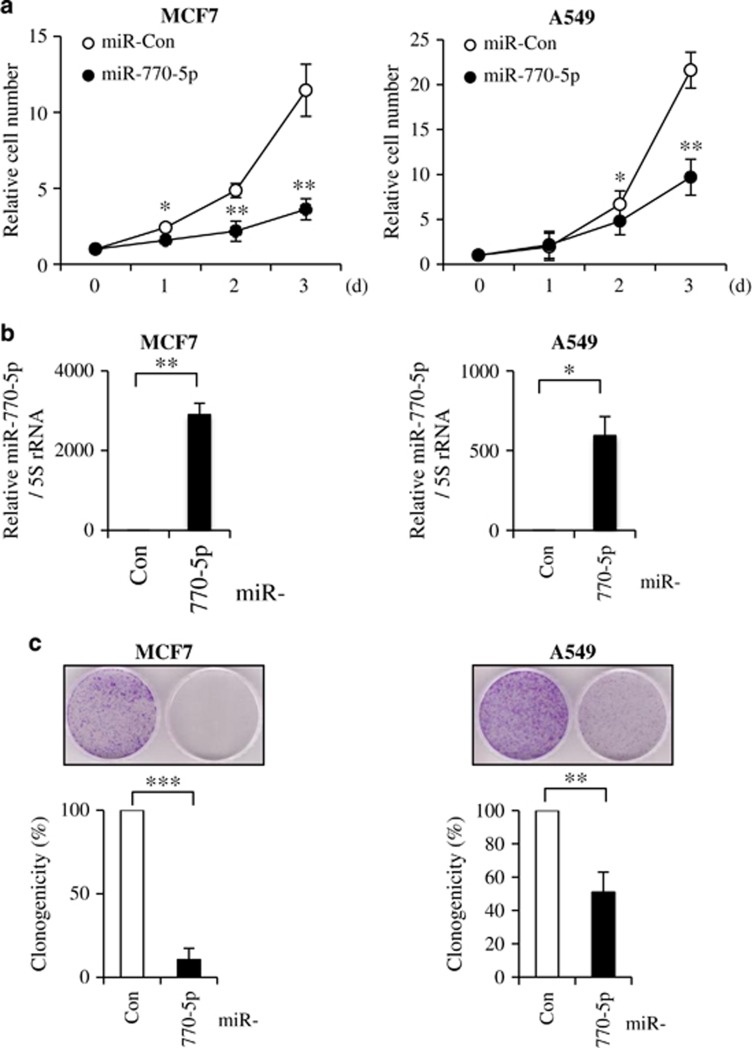
MiR-770-5p suppresses cancer cell survival. MCF7 and A549 cells were transfected with either miR-Con or miR-770-5p mimic. (**a**) Cell numbers were counted at indicated time intervals after transfection. Cell numbers at 0 day was set as 1 (1.5 × 10^4^ cells) and relative cell numbers were calculated (**b**) Levels of miR-770-5p were examined with qRT-PCR at 3 days after transfection. 5S rRNA was used as internal control. (**c**) Clonogenicities of miR-770-5p-transfected cells were analyzed at 7 days after transfection. Each bars represent means (*n* = 3) and S.D. (***P<0.001, **P<0.01, *P<0.05)

To identify novel IR-responsive miRNAs, we irradiated MCF7 human breast carcinoma cells with 6 Gy of IR. Total RNAs from irradiated cells were isolated at each time point (0, 2, 8, and 24 h), and analyzed miRNA expression changes by using of miRNA microarray analysis. IR-responsive miRNA profiles were normalized and ranked according to fold changes. Heat maps of the top 20 up and downregulated miRNAs in IR-exposed MCF7 cells are shown in Figures 1a and b. The extended data sets of 229 IR-responsive miRNAs are shown in Supplementary Figure S1. Among them, miR-770-5p, miR-1287, and miR-371-5p exhibited evident induction at every time point post irradiation (2, 8, and 24 h) (Figure 1a). We assessed level changes of miR-770-5p, miR-1287, and miR-371-5p at 2, 8, and 24 h post irradiation by qRT-PCR in MCF7 cells (Figure 1c). Since there is no report regarding the cellular function of miR-770-5p, which was evidently induced by IR exposure, we decided to explore the roles of miR-770-5p and its target in the radiation response. First, we transfected miR-770-5p mimic into MCF7 cells, and examined relative cell number and clonogenicity. Relative cell numbers in miR-770-5p-transfected cells evidently decreased compared to those in miR-control (Con)-transfected cells (Figure 2a). MiR-770-5p-transfected MCF7 cells showed poor clonogenicity (Figure 2c). When we examined the transfection effect of miR-770-5p in A549 human lung adenocarcinoma cells, relative cell numbers and clonogenicity also evidently decreased compared to those in miR-Con-transfected cells (Figures 2a and c). Increased levels of miR-770-5p were confirmed with qRT-PCR at 3 days after transfection in MCF7 and A549 cells (Figure 2b).

### Ectopic expression of miR-770-5p *per se* induces apoptosis

**Figure 3:**
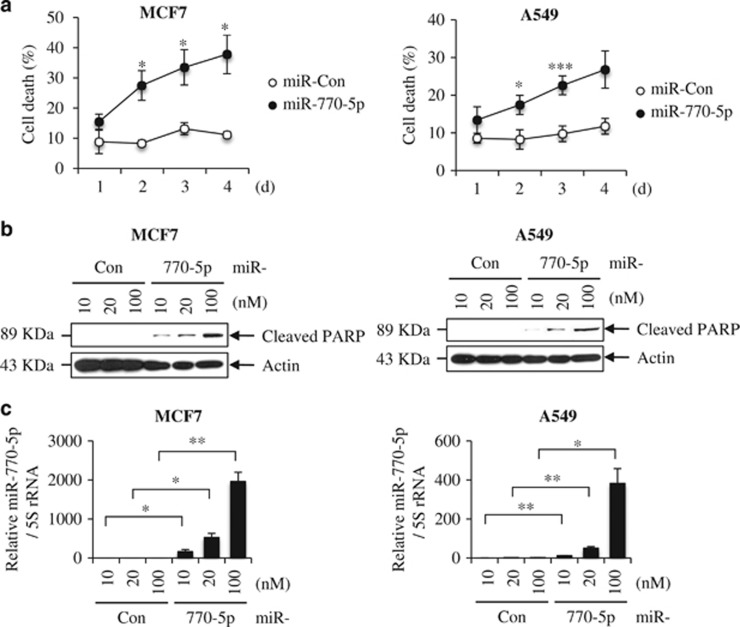
MiR-770-5p *per se* induces apoptotic cell death. MCF7 and A549 cells were transfected with either miR-Con or miR-770-5p mimic. (**a**) Percentage of cell death was analyzed at the indicated time intervals after transfection of miR-770-5p by Trypan Blue exclusion analysis. Each bars represent means (*n*=3) and S.D. (***P<0.001, **P<0.01, *P<0.05). (**b**) After transfection of miR-770-5p at the indicated concentrations, cleaved PARP level was analyzed in MCF7 and A549 cells by western blotting. Actin was used as a loading control. (**c**) qRT-PCR shows the relative levels of miR-770-5p at the indicated concentrations of miR-Con and miR-770-5p. 5S rRNA was used as internal control

Since we observed profound decreases in relative cell number, and clonogenicity in miR-770-5p-transfected MCF7 and A549 cells, we examined whether or not miR-770-5p could affect cell death. Trypan Blue exclusion assay showed that ectopic expression of miR-770-5p induced cell death in both MCF7 and A549 cells compared to that in miR-Con-transfected cells (Figure 3a). We detected an increase in PARP cleavage, a general marker of apoptosis, in a dose-dependent manner of miR-770-5p in both MCF7 and A549 cells at 72 h after transfection (Figure 3b). MiR-770-5p levels were increased in dose-dependent manner in miR-770-5p-transfected MCF7 and A549 cells (Figure 3c).

### PBK is a direct target of miR-770-5p

**Figure 4:**
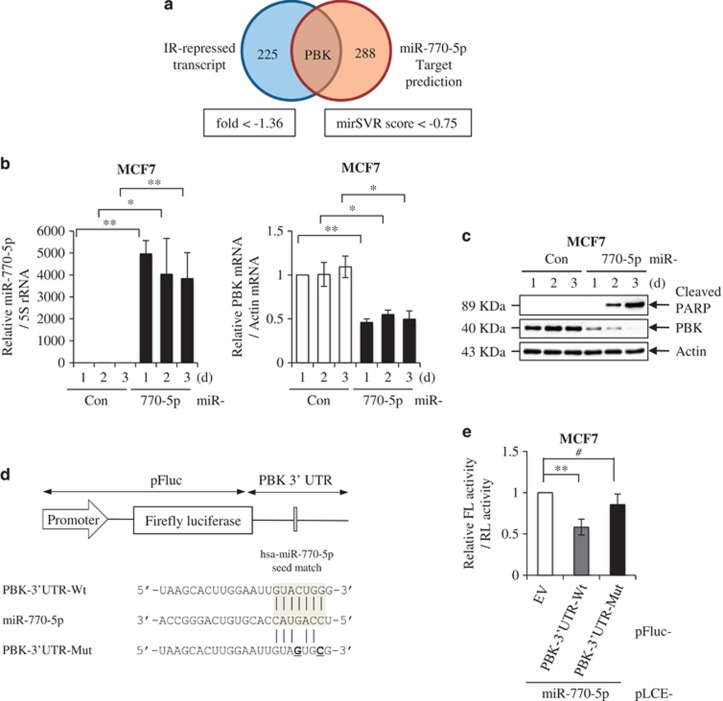
PBK is a direct target of miR-770-5p. (**a**) Candidate genes as targets for miR-770-5p were predicted using *RNA* sequencing analysis and based on miRSVR score. Among 225 IR-repressed genes, only PBK had a mirSVR score for miR-770-5 <−0.75. (**b**) Level changes in miR-770-5p and PBK mRNA were observed by qRT-PCR at the indicated time intervals after transfection of either miR-Con or miR-770-5p in MCF7 cells. 5S rRNA and actin mRNA was used as internal control, respectively. (**c**) Level changes in PBK protein and cleaved PARP were observed at the indicated time intervals after transfection of either miR-Con or miR-770-5p in MCF7 cells. Actin was used as a loading control. (**d**) Reporter gene construct containing a putative binding site for miR-770-5p (PBK-3′UTR-Wt) and its mutated sequences (PBK-3′UTR-Mut) in the 3′UTR of PBK. (**e**) MCF7 cells were co-transfected with either PBK-3′UTR-Wt or PBK-3′UTR-Mut with miR-770-5p, after which relative luciferase activities were analyzed at 2 days after transfection. Each bars represent means (*n* = 3) and S.D. (**P<0.01, *P<0.05, ^#^*P*>0.05)

To identify potential targets of miR-770-5p in miR-770-5p-mediated apoptosis, we analyzed our previous expression array data reflecting changes in global mRNA expression profiles upon IR exposure.^18^ We obtained 225 IR-repressed genes showing fold changes of <−1.36 (log2) from the expression array analysis (Figure 4a and Supplementary Table S1). We also used miRSVR score^19^ to predict potential miR-770-5p targets based on the microRNA.org website (www.microrna.org). The 288 candidate targets were selected based on a miRSVR score threshold of <−0.75 (Figure 4a and Supplementary Table S2). From the IR-responsive expression profile and bioinformatics analyses, we finally identified PBK as a strong candidate since it was both highly repressed by IR and shown to contain a target sequence for miR-770-5p in its 3′UTR. To verify whether or not miR-770-5p regulates PBK expression, we evaluated changes in PBK level after transfection of miR-770-5p. After confirmation of increased miR-770-5p levels, we examined PBK mRNA and protein levels in miR-770-5p-transfected cells (Figures 4b and c). MiR-770-5p evidently reduced the PBK mRNA level (Figure 4b, right). MiR-770-5p also reduced the level of PBK protein, and PBK protein level was closely correlated with PARP cleavage (Figure 4c). We also examined PBK level and PARP cleavage in miR-770-5p-transfected HCT116 human colon carcinoma cells (Supplementary Figure S2). Levels of PBK mRNA and protein were evidently decreased and PARP cleavage was observed in miR-770-5p-transfected HCT116 cells (Supplementary Figure S2). PBK harbors a miR-770-5p seed-matched sequence in its 3′UTR, as illustrated in Figure 4d. We next generated reporters containing the firefly luciferase gene followed by either wild-type or mutated PBK-3′UTR in the putative miR-770-5p-binding site (PBK-3′UTR-Wt or PBK-3′UTR-Mut) (Figure 4d). Each firefly luciferase (FL) construct was co-transfected with internal control *Renilla* luciferase (RL) in the presence or absence of miR-770-5p, after which FL activity was measured and normalized based on RL activity. PBK-3′UTR-Wt reporter activity was reduced compared to the reporter control, whereas PBK-3′UTR-Mut reporter activity was not significantly altered in miR-770-5p-transfected cells (Figure 4e). These data demonstrate that miR-770-5p negatively regulates PBK expression through direct binding to its 3′UTR.

### MiR-770-5p sensitizes tumor cells to radiation

**Figure 5:**
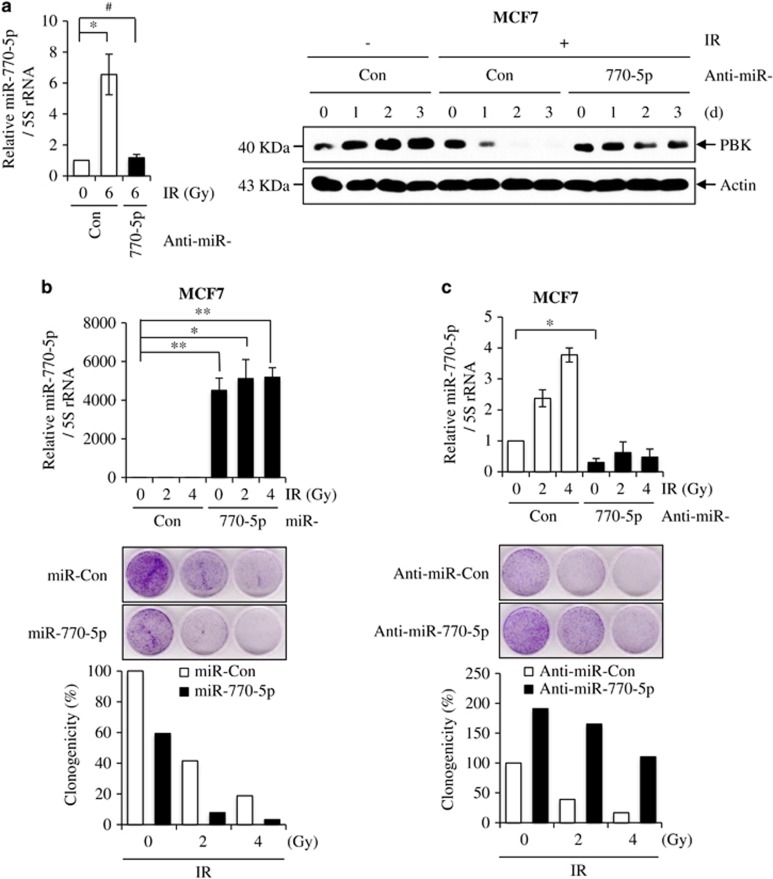
MiR-770-5p sensitizes cancer cells to radiation. (**a**) MCF7 cells were transfected with either anti-miR-Con or anti-miR-770-5p, followed by exposure to 6 Gy of IR. Levels of miR-770-5p was analyzed at 2 days after irradiation by qRT-PCR. Levels of PBK protein were analyzed by western blotting at the indicated time intervals. 5S rRNA and actin were used as internal controls of qRT-PCR and western blotting, respectively. (**b**) MCF7 cells were transfected with either miR-Con or miR-770-5p, followed by exposure to indicated doses of IR. The relative level of miR-770-5p was confirmed by using qRT-PCR (top). 5S rRNA was used as an internal control. Cell viability was analyzed by clonogenic assay (middle) and represents as a graph (bottom). Each bar in the graph indicates the mean of two independent experiments. (**c**) MCF7 cells were transfected with either anti-miR-Con or anti-miR-770-5p, followed by exposure to the indicated doses of IR. Decreased level of miR-770-5p was measured by qRT-PCR (top). 5S rRNA was used as an internal control. Cell viability was analyzed by clonogenic assay (middle) and represents as a graph (bottom). Each bar in the graph indicates the mean of two independent experiments. Each bars represent means (*n* = 3) and S.D. (**P<0.01, *P<0.05, ^#^*P*>0.05)

To examine the physiological relationship between miR-770-5p and PBK in response to IR, we irradiated anti-miR-Con- and anti-miR-770-5p-transfected MCF7 cells and then examined level changes in PBK expression. IR treatment drastically reduced the amount of PBK protein in anti-miR-Con-transfected cells (Figure 5a). In contrast, anti-770-5p-transfected cells showed restored expression levels of PBK post irradiation (Figure 5a), suggesting that miR-770-5p is a key factor in the regulation of PBK expression upon IR exposure. Next, MCF7 cells were transfected with either miR-770-5p or anti-miR-770-5p, followed by IR exposure at the indicated doses, as shown in Figures 5b and c. Whereas miR-770-5p further reduced clonogenic ability, anti-miR-770-5p increased clonogenic ability post irradiation (Figures 5b and c). These data demonstrate that miR-770-5p plays a critical role in enhancing radiosensitivity via PBK regulation in tumor cells. Effects of miR-770-5p and anti-miR-770-5p transfection on intracellular miR-770-5p levels were validated in non-irradiated and irradiated MCF7 cells (Figures 5a–c). Our findings suggest that IR-induced miR-770-5p potentiates IR sensitivity by promoting apoptotic cell death.

### Reconstitution of PBK rescues tumor cells from miR-770-5p-induced cell death

**Figure 6:**
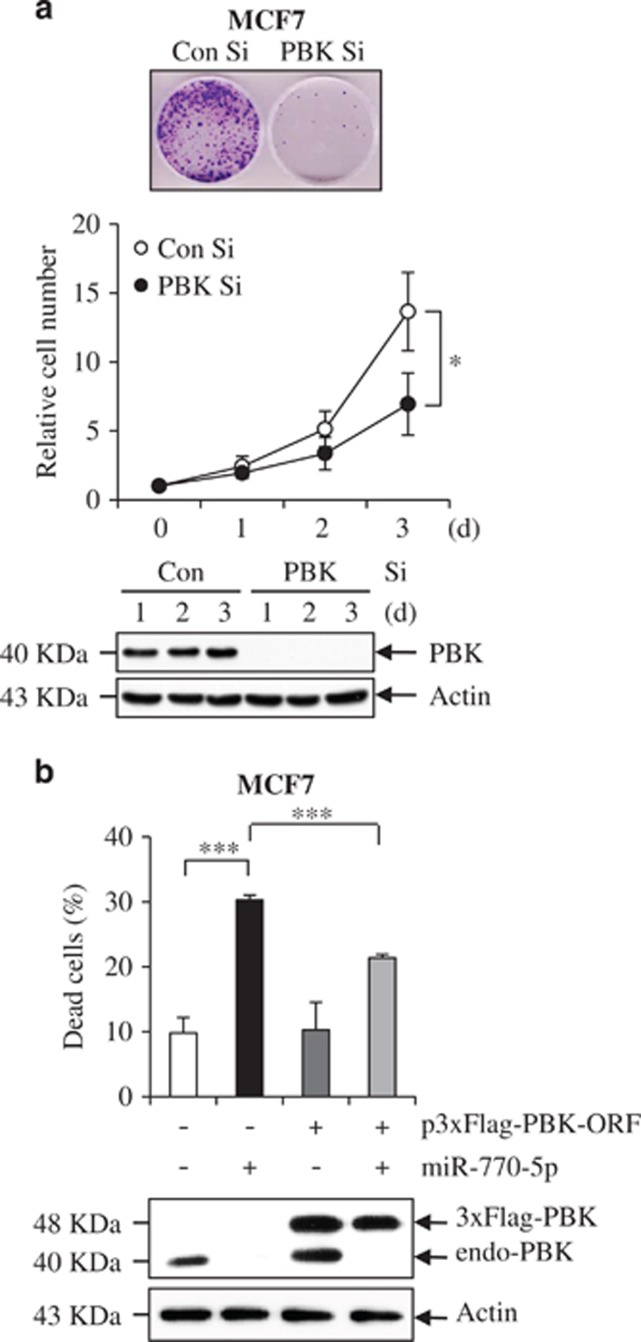
MiR-770-5p induces apoptosis through PBK downregulation. (**a**) After transfection of either Con Si or PBK Si, clonogenicity (top) and relative cell numbers (middle) were analyzed at 7 days as well as at the indicated time intervals, respectively. The depletion of PBK level was confirmed by western blotting (bottom). Actin was used as a loading control. (**b**) MCF7 cells were transfected with either p3 × Flag-EV or p3 × Flag-PBK-ORF construct, after which the effect of ectopic expression of PBK on miR-770-5p-induced cell death was analyzed by Trypan Blue exclusion assay at 2 days after transfection (top). Endogenous and exogenous PBK levels were analyzed by western blotting (bottom). Actin was used as a loading control. Each bars represent means (*n* = 3) and S.D. (***P<0.001, *P<0.05)

We also examined the effect of PBK knockdown using short interfering RNA on MCF7 cell survival. PBK depletion suppressed clonogenicity and reduced relative cell numbers (Figure 6a). Because of the existence of multiple targets for a single miRNA,^11^ we examined whether or not miR-770-5p exerts a proapoptotic effect through targeting of PBK. Flag-tagged PBK lacking its 3′UTR containing the target region of miR-770-5p (p3 × Flag-PBK-ORF) was generated and co-transfected with miR-770-5p into MCF7 cells. When we performed immunoblot analysis, miR-770-5p evidently reduced the level of endogenous PBK, but had no effect on PBK expression from p3 × Flag-PBK-ORF (Figure 6b, bottom). MiR-770-5p-induced cell death was evidently reduced by ectopic expression of p3 × Flag-PBK-ORF (Figure 6b, top). These results indicate that miR-770-5p potentiates cell viability through direct targeting of PBK. Taken together, our data suggest that IR-responsive miR-770-5p plays a role in induction of apoptosis via regulation of PBK expression. Thus, further increasing the miR-770-5p level in IR-exposed cells can likewise increase the radiosensitivity of tumor cells to IR.

### MiR-770-5p retards tumor growth in a xenograft tumor mouse model through PBK targeting

**Figure 7:**
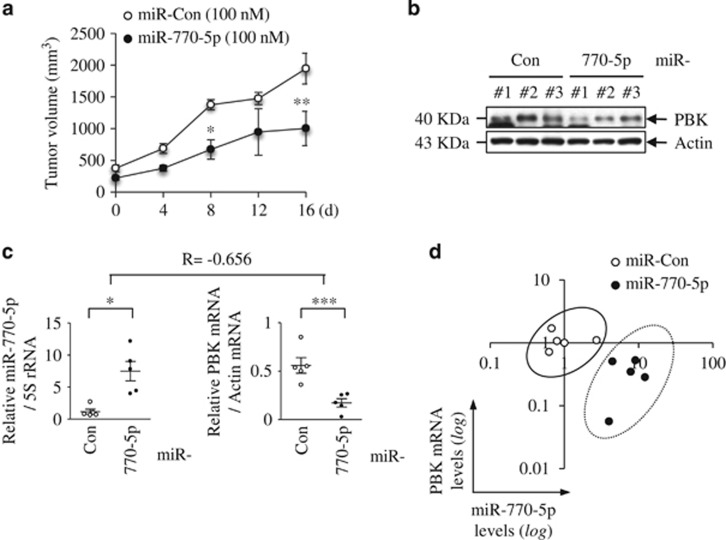
MiR-770-5p retards tumor growth due to suppression of PBK expression in a xenograft tumor mouse model. (**a**) Tumor volume in xenograft mice (*n*=5) was measured at the indicated times. (**b**) Western blot analysis of PBK protein level in tumor tissues. Actin was used as a loading control. (**c**) qRT-PCR analysis of miR-770-5p and PBK mRNA in tumor tissues (*n*=5). Expression levels were normalized to 5S rRNA and actin mRNA, respectively. Correlation between miR-770-5p and PBK mRNA was analyzed using the Pearson correlation coefficient (*R*). (**d**) *In vivo* correlation between miR-770-5p and PBK mRNA. Normalized miR-770-5p and PBK mRNA levels from tumor samples were plotted on logarithmic scales. Error bars indicate S.E.M. (***P<0.001, **P<0.01, *P<0.05)

To explore the biological significance of our *in vitro* observations, we examined the role of miR-770-5p in a xenograft tumor mouse model. We found that the growth rate of xenografted tumors was significantly retarded in mice injected subcutaneously with miR-770-5p (100 nM)-transfected cells compared to mice injected with miR-Con-transfected cells (Figure 7a). We tracked the extent of PBK expression in tumor tissues injected with miR-770-5p- and miR-Con-transfected cells. Western blot analysis demonstrated that PBK expression was attenuated in tumor tissues bearing miR-770-5p (Figure 7b). To confirm that reduction of PBK expression was due to the effects of miR-770-5p transfection *in vivo*, we analyzed miR-770-5p and PBK mRNA levels using quantitative reverse transcription-PCR (qRT-PCR) in tumor tissues (Figure 7c). Correlation between miR-770-5p and PBK mRNA levels in tumor tissues was normalized and assessed using the Pearson correlation coefficient, and the *R*-value indicated a negative correlation between miR-770-5p and PBK expression (*R*=−0.656). Plot of the miR-770-5p and PBK mRNA levels from each tumor tissue showed distinct clustering (Figure 7d). These data indicate that miR-770-5p suppresses PBK expression, resulting in tumor growth retardation.

**Figure 8:**
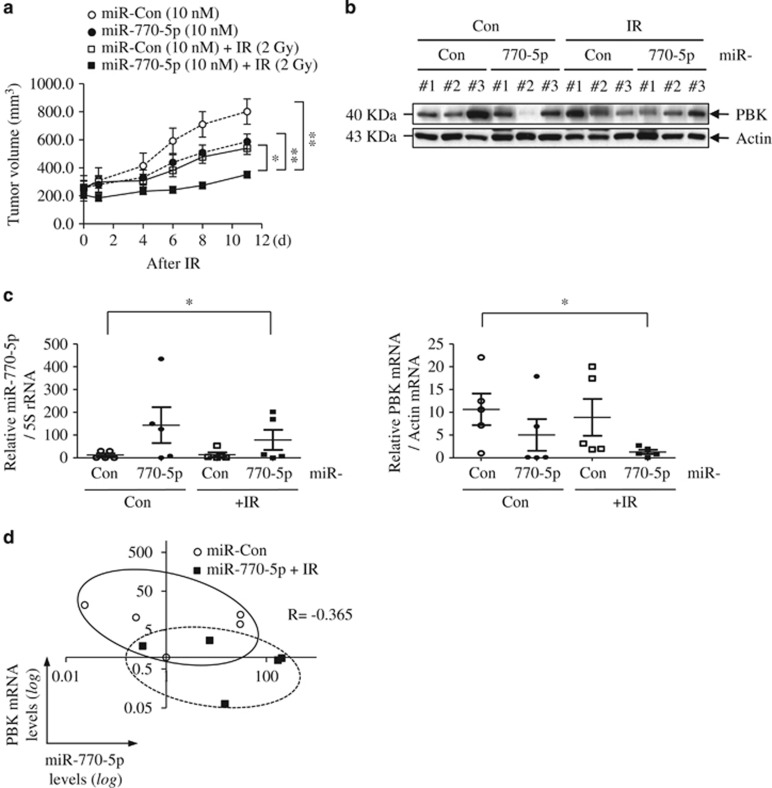
MiR-770-5p sensitizes tumors exposed to 2 Gy of IR in a xenograft tumor mouse model. (**a**) Tumor volume in xenograft mice (*n*=9) was measured at the indicated times. (**b**) Western blot analysis of PBK protein level in tumor tissues. Actin was used as a loading control. (**c**) qRT-PCR analysis of miR-770-5p and PBK mRNA in xenografted tumor tissues (*n*=5). Expression levels were normalized to 5S rRNA and actin mRNA, respectively. (**d**) *In vivo* correlation between miR-770-5p and PBK mRNA. Normalized miR-770-5p and PBK mRNA levels from tumor samples were plotted on logarithmic scales. Correlation between miR-770-5p and PBK mRNA was analyzed using the Pearson correlation coefficient (*R*). Each bars indicate S.E.M. (**P<0.01, *P<0.05)

Next, since we wanted to know whether miR-770-5p could sensitize tumors to IR *in vivo*, we developed a xenograft tumor mouse model and allowed to form tumor. And then, we injected 10 nM of miR-770-5p to the whole tumor mass and locally exposed 2 Gy of IR to xenograft tumor 1 day after injection. Combination treatment group (miR-770-5p+IR) significantly suppressed tumor growth compared to other groups of mice (miR-Con, miR-770-5p, and miR-Con+IR) (Figure 8a). In combination treatment group, we observed significant decrease of PBK mRNA and attenuated expression of PBK protein (Figures 8b and c). Negative correlation between miR-770-5p and PBK (*R*=−0.356) was appeared as shown in Figure 8d. Since we applied low concentration of miR-770-5p (10 nM) and low dose of IR (2 Gy) once to evaluate the combination effect of mIR-770-5p and IR, no marked change of either miR-770-5p or PBK expression was observed in either IR- or miR-770-5p-treated groups (Figures 8b and c). These data indicate that miR-770-5p sensitized tumors to radiotherapy through the suppression of PBK expression. Collectively, our results suggest that miR-770-5p could be a potential target for the radiotherapy.

## Discussion

Great technological progress in radiation therapy has been achieved over the last 20 years, and the ability to cure a wide range of malignancies has been attained. Research in radiation biology is currently focused on increased radiotherapy efficacy. To approach this goal, we have to better understand the cellular response to IR exposure. Each cell determines whether the cell will die, repair the damage, or proceed through cellular division despite the damage after a critical period from radiation exposure.^20^ On the molecular level, radiation-induced damage initiates a complex signaling cascade in cells, resulting in a variety of responses that include cell cycle arrest, induction of stress-response genes, DNA repair, and apoptosis.^21^ IR damage activates a number of stress-response-signaling pathways that contribute to lethality, and many of these may be independent of DNA damage.^20, 21^ Radiation can also up or downregulate miRNA expression, and such alteration of miRNA expression profiles could affect the final outcome of radiation therapy.

MiRNAs are found in most eukaryotes and are involved in many important cellular responses.^22^ It has recently been shown that expression levels of miRNAs vary significantly after IR exposure.^2, 9, 13^ The IR-induced DDR modulates miRNA expression and biogenesis. In addition, Mao *et al.*^23^ and Marta *et al.*^21^ very recently reviewed IR-responsive miRNAs and their targets in various cell lines. Regulation of miRNAs was shown to influence cellular sensitivity to radiation, primarily through modulation of molecules involved in the DDR such as ATM, ATR, RAD51, and DNA-PK.^2, 9, 13^ In particular, miR-421 suppresses ATM expression and increases sensitivity to IR.^15^ MiR-214 is upregulated in radio-resistant non-small cell lung cancer (NSCLC) cells relative to their radiosensitive counterparts.^24^ Overexpression of miR-214 in radiosensitive NSCLC cells protects against RT-induced apoptosis via downregulation of p38 kinase. Elevation of miR-185, which is downregulated in response to IR sensitizes renal cell carcinoma cells to IR both *in vitro* and *in vivo* by targeting ATR kinase.^16^ The p53-responsive miRNA miR-34a binds to the 3′UTR of RAD51 and inhibits double-strand break repair in NSCLC cells.^14^

Until now, three papers regarding miR-770-5p have been reported. Specifically, miR-770-5p was shown to be highly expressed in type 2 diabetes mellitus patients,^24^ whereas miR-770-5p was found to be downregulated in a rat model of temporal lobe epilepsy compared with the control group.^22^ Zhao *et al.*^25^ reported very recently that miR-770-5p expression served as a prognostic biomarker and overexpression of miR-770-5p reduced survival in cisplatin-treated ovarian cancer cells. They showed that miR-770-5p inhibits cisplatin chemoresistance by targeting ERCC2 *in vivo* and *in vitro* in human ovarian cancer. In this study, we identified miR-770-5p as an upregulated miRNA in response to IR. Overexpression of miR-770-5p induced apoptosis, and elevation of miR-770-5p sensitized MCF7 breast carcinoma and A549 lung carcinomas to IR through direct targeting of PBK. Ectopic expression of miR-770-5p in tumor tissue exhibited retarded tumor growth in xenograft model mice. We also demonstrated an inverse correlation between miR-770-5p and PBK both *in vitro* and *in vivo*. PBK was identified as an upregulated serine–threonine kinase in Burkitt's lymphoma cell lines.^26, 27, 28^ PBK was further shown to be upregulated in rapidly proliferating cells as well as a variety of tumor.^29, 30, 31^ PBK was shown to promote transformation,^28^ and knockdown of PBK reduced tumorigenic and metastatic properties both *in vivo* and *in vitro*.^32, 33, 34^ Many studies have also strongly demonstrated that PBK inhibition might be beneficial for tumor regression and a good prognosis.^35, 36, 37^ Consistent with the role of PBK in previous studies, this study suggests that PBK could be a useful therapeutic target for a sensitized IR response and could be achieved through ectopic expression of miR-770-5p. We searched deep sequencing online data to demonstrate correlation between miR-770-5p and PBK in human tissues (miRGator.kobic.re.kr). As based on small RNA-seq from ENCODE Database, we identified strong negative correlation between miR-770-5p and PBK (correlation coefficient (*r*) value was −0.67). In analysis from TCGA-breast invasive carcinoma Database, *r*-value between miR-770-5p and PBK was −0.31. We believe that these analysis data further support our conclusion.

The field of miRNA study is currently one of the fastest growing research fields. The involvement of miRNAs in important biological processes and diseases underlines their importance. Specifically, the diagnostic and prognostic value of miRNAs in cancer cannot be overemphasized. We suggest that miR-770-5p is a promising agent to improve the efficacy of cancer radiotherapy. In general, many miRNAs modulate radiosensitivity by targeting the DDR. However, we showed that miR-770-5p renders radiosensitivity through blockade of the PBK signaling pathway. Despite numerous studies regarding the oncogenic potential of PBK, we report here for the first time that miR-770-5p can directly target PBK in the radiation response. Alteration of miR-770-5p expression upon IR can be used not only as a promising method for improving the efficacy of cancer radiotherapy, but also as a biomarker for IR exposure.

## Materials and methods

### Cell culture

MCF7 cells were cultured in Dulbecco's Modified Eagle's Medium (DMEM; WelGENE, Daegu, Korea), A549 cells were cultured in RPMI 1640 (WelGENE), and HCT116 cells were cultured in McCoy's 5A medium (WelGENE) at 37 °C in a 5% CO_2_ incubator. Cell culture medium was supplemented with 10% fetal bovine serum (Lonza, Basel, Switzerland), 1% penicillin, and streptomycin solution (WelGENE).

### Plasmid construction

Plasmid expressing hsa-miR-770-5p was generated using genomic DNA from A549 cell lines as a template, as well as 5′-atgcctcgagAAAGTGGGGTGCTCAGGAAT-3′ and 5′-atgcgcggccgcGGAGAAGCTTCAGCAGGTGT-3′ as primers. Amplified PCR products were inserted between the XhoI and NotI sites of pLCE vector.^37^ To investigate the direct effect of miR-770-5p on the 3′ UTR of PBK (PBK-3′UTR), we amplified the 3′UTR of PBK from A549 cDNA libraries by PCR using 5′-atgcctcgagTGATCATCTCAGCTGAAGTG-3′ and 5′-atgcgcggccgcTTGTACTGTACAAAGTGCTA-3′ primer pairs, and the resulting products were ligated into the 3′UTR of the firefly luciferase (*Fluc*) reporter gene. The Fluc-PBK-3′UTR reporter plasmid (pFluc-PBK-3′UTR-Wt) was sequenced. pFluc-PBK-3′UTR-Mut harboring miR-770-5p seed region mutations (GTACTGG to GTAGTGC) was generated from pFluc-PBK-3′UTR-WT as a template with 5′-GCACTTFFAATTGTAGTGCGTTTTCTGTAAAG-3′ and 5′-CTTTACAGAAAACGCACTACAATTCCAAGTGC-3′ using a QuikChange Site-Directed Mutagenesis Kit (Agilent Technologies, Santa Clara, CA, USA).

### Irradiation

Cells were irradiated to *γ*-ray using a ^137^Cs *γ*-ray source (Atomic Energy of Canada Ltd, Mississauga, Ontario, Canada) at a dose rate of 3.2 Gy per min. Cells were exposed using the indicated single irradiation dose (6 Gy) and then collected at the indicated times in each experiment.

### MiRNA array analysis

MiRNA-enriched total RNA from MCF7 cells exposed to 6 Gy of IR was extracted using TRI reagent (Molecular Research Center, Cincinnati, OH, USA) following the manufacturer's protocol. Samples of miRNA-enriched total RNA were labeled and hybridized using a miRNA Labeling Reagent and Hybridization Kit (Agilent, Santa Clara, CA, USA) according to the manufacturer's protocol. After hybridization, the miRNA microarray was washed in GE Wash Buffer 1 (Agilent) and GE Wash Buffer 2 (Agilent) for 5 min in turn. The slides were then scanned with Agilent Microarray Scanner (Agilent). The microarray images were analyzed with Feature Extraction Software (Agilent). MiRNAs were considered as significantly differentially expressed if their ratios were >2.0 or <0.5.

### qRT-PCR analysis

Total RNA was isolated and subjected to reverse transcription using RevertAid H Minus Reverse Transcriptase (Thermo Fisher Scientific, Waltham, MA, USA) according to the manufacturer's recommendations. MiRNAs were isolated and subjected to reverse transcription using a GenoExplorer miRNA First-Strand cDNA Core Kit (GenoSensor Corporation, Tempe, AZ, USA) according to the manufacturer's instructions. The qRT-PCR was performed using iQ SYBR Green Supermix (Bio-Rad, Hercules, CA, USA) on Chromo4 Real-Time PCR Detection System (Bio-Rad) with mRNA-specific primers: for PBK, 5′-TCTCATTCTCCTTGGGCTGT-3′ (sense) and 5′-AAAGGATCTTGGCTGGCTTT-3′ (antisense); and for actin, 5′-CAAGAGATGGCCACGGCT-3′ (sense) and 5′-TCCTTCTGCATCCTGTCGGC-3′ (antisense). The miRNA-specific primers were purchased from GenoSensor Corp. The relative fold change of RNA expression values was normalized to that of an internal control (5S rRNA or actin mRNA).

### Immunoblot analysis

Cells lysates were prepared in RIPA lysis buffer (50 mM Tris-HCl, pH 8.0, 150 mM NaCl, 2 mM EDTA, 1% Triton X-100, 0.5% sodium deoxycholate, 0.1% SDS) containing protease inhibitor (Roche, Indianapolis, IN, USA). Before electrophoresis, lysates were mixed with 2 × Laemmli sample buffer and boiled. The protein concentration was determined using the Bio-Rad Protein Assay kit according to manufacturer's protocol (Bio-Rad). Equal amounts of protein (30 *μ*g) were fractionated by SDS-PAGE and then transferred to a nitrocellulose membrane. The nitrocellulose membrane was blocked with 5% non-fat milk and incubated with primary antibodies at 4 °C overnight. Then, blots were incubated with horseradish peroxidase-conjugated goat anti-mouse and goat anti-rabbit antibodies. Protein detection was conducted with ECL reagents (Thermo Scientific, Waltham, MA, USA).

### Dual luciferase reporter assay

Luciferase reporter assay was performed as previously described.^33^ RL reporter control plasmid (pRluc) was co-transfected into 293 T cells with pFluc, pFluc-PBK-3′UTR-Wt, or pFluc-PBK-3′UTR-Mut, together with miR-770-5p-expressing plasmid using FuGENE6 (Promega, Madison, WI, USA). FL and RL activities were evaluated by using the Dual Luciferase Reporter Assay System (Promega) and VICTOR X Light plate reader (PerkinElmer), after which FL activity was normalized by RL activity.

### Transfection

Cells were transfected with miR-770-5p mimic or mimic control (GenoExplorer) using Lipofectamine RNAiMAX (Invitrogen, Karlsruhe, Germany). In the same way, cells were transfected with either anti-miR-770-5p or anti-miR control. Transfection of plasmids was carried out using Lipofectamine 2000 reagent (Invitrogen) according to the manufacturer's instructions.

### Cell growth rate and colony-forming assay

Cell growth rate was determined by counting viable cells after staining with Trypan Blue (0.4% GIBCO, Grand Island, NY, USA). Viable (unstained) and dead (stained) cells were counted by hemocytometer microscopy. Clonogenicity was examined using colony-forming assay. Approximately 1 × 10^4^ cells were seeded on 60 mm plates and cultured for 7 days. Colonies were stained with Diff-Quick (Sysmex, Kobe, Japan).

### Xenograft mice model experiment

For xenograft mice model experiment to examine effect of miR-770-5p on tumor growth, HCT116 cells (5 × 10^6^) transfected with 100 nM of miR-Con or miR-770-5p were injected subcutaneously into the lateral hind leg of 6-week-old immunodeficient BALB/c female nude mice (nu/nu; Orient Bio Inc., Seongnam, Korea) for each group (*n*=5). Tumor growth was monitored periodically for 16 days. For xenograft mice model experiment to examine combination effect of miR-770-5p and IR on tumor growth, HCT116 cells (5 × 10^6^) were injected subcutaneously into the lateral hind leg of 6-week-old immunodeficient BALB/c female nude mice (nu/nu; Orient Bio Inc.) for each group (*n*=9). When the tumor reached an average volume of ~200 mm^3^, 10 nM of miR-Con or miR-770-5p with AteloGenes Local Use (KOKEN, Tokyo, Japan) was injected to wrap up the whole tumor mass. Xenografted tumors were locally exposed to IR of 2 Gy 1 day later. Local regional irradiation of xenografted tumor was performed under anesthesia using X-RAD 320 irradiator (Precision X-ray Ltd, North Branford, CT, USA). Tumor growth was monitored periodically for 12 days. Average tumor volume (V) was determined as (L × W^2^)/2; measurements of tumor length (*L*) and width (*W*) were taken with a caliper. All animal studies were conducted with the approval of the Institutional Animal Care and Use Committee of the Korea Institute Radiological and Medical Sciences (approval nos KIRAMS 2014-0017 and KIRAMS 2016-0064).

### Statistical analysis

Differences between the experimental groups were calculated using Student's two-tailed *t*-test. *P*-values of <0.05 were considered to be statistically significant. Correlation between PBK mRNA and miR-770-5p levels in tumors was assessed using Pearson's correlation coefficient analysis.
