# Supplementary material for: Radiation-inducible miR-770-5p sensitizes tumors to radiation through direct targeting of PDZ-binding kinase
Source: Cell Death Dis. 2017 Mar 23;8(3):e2693–. doi: 10.1038/cddis.2017.116 (PMC5386522; doi:10.1038/cddis.2017.116)
Supplement: Supplementary Figures and Tables [file cddis2017116x1.ppt]

## Slide 1
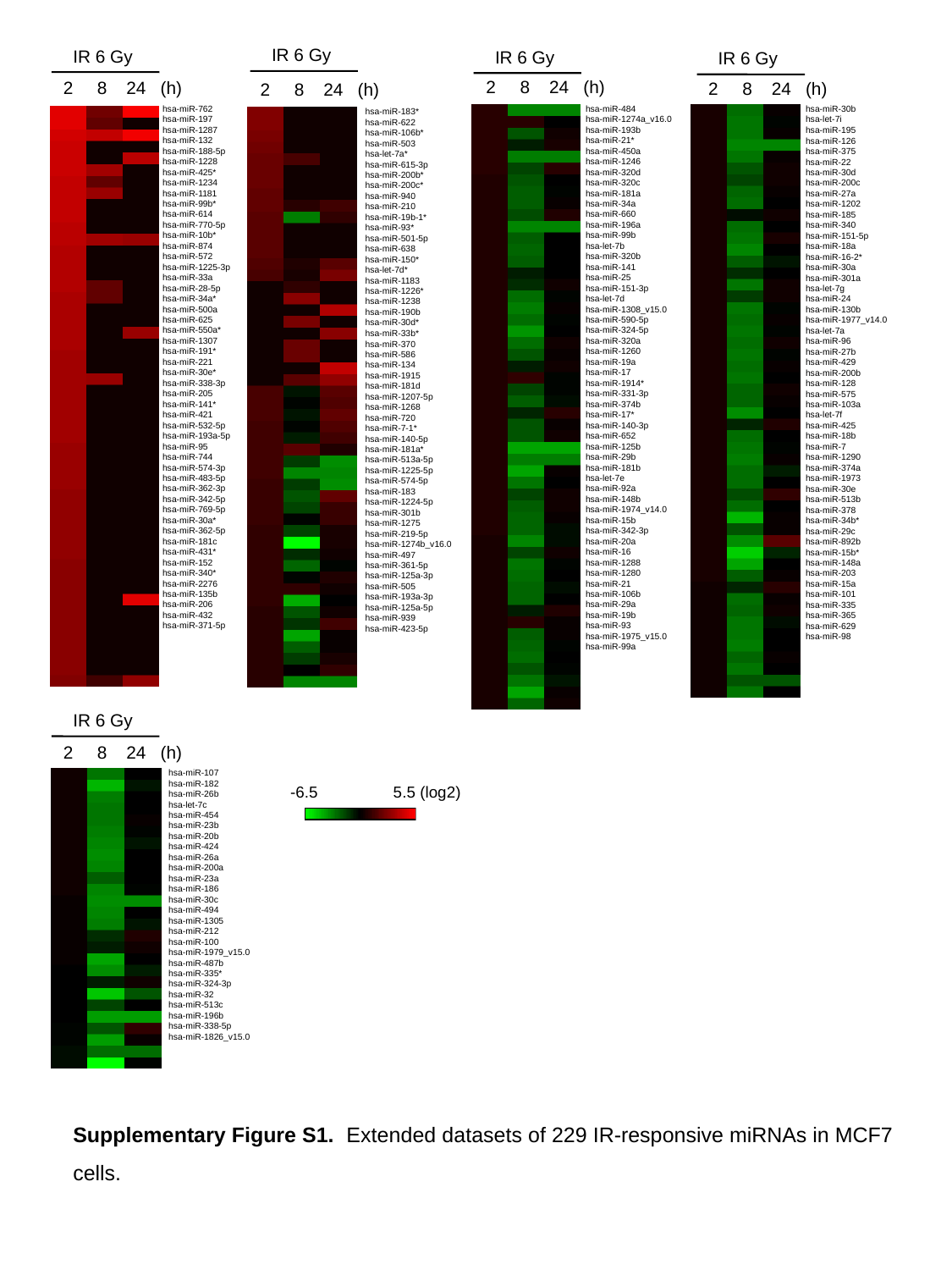

IR 6 Gy
IR 6 Gy
IR 6 Gy
IR 6 Gy
2 8 24 (h)
2 8 24 (h)
2 8 24 (h)
2 8 24 (h)
hsa-miR-762
hsa-miR-197
hsa-miR-1287
hsa-miR-132
hsa-miR-188-5p
hsa-miR-1228
hsa-miR-425*
hsa-miR-1234
hsa-miR-1181
hsa-miR-99b*
hsa-miR-614
hsa-miR-770-5p
hsa-miR-10b*
hsa-miR-874
hsa-miR-572
hsa-miR-1225-3p
hsa-miR-33a
hsa-miR-28-5p
hsa-miR-34a*
hsa-miR-500a
hsa-miR-625
hsa-miR-550a*
hsa-miR-1307
hsa-miR-191*
hsa-miR-221
hsa-miR-30e*
hsa-miR-338-3p
hsa-miR-205
hsa-miR-141*
hsa-miR-421
hsa-miR-532-5p
hsa-miR-193a-5p
hsa-miR-95
hsa-miR-744
hsa-miR-574-3p
hsa-miR-483-5p
hsa-miR-362-3p
hsa-miR-342-5p
hsa-miR-769-5p
hsa-miR-30a*
hsa-miR-362-5p
hsa-miR-181c
hsa-miR-431*
hsa-miR-152
hsa-miR-340*
hsa-miR-2276
hsa-miR-135b
hsa-miR-206
hsa-miR-432
hsa-miR-371-5p
hsa-miR-484
hsa-miR-1274a_v16.0
hsa-miR-193b
hsa-miR-21*
hsa-miR-450a
hsa-miR-1246
hsa-miR-320d
hsa-miR-320c
hsa-miR-181a
hsa-miR-34a
hsa-miR-660
hsa-miR-196a
hsa-miR-99b
hsa-let-7b
hsa-miR-320b
hsa-miR-141
hsa-miR-25
hsa-miR-151-3p
hsa-let-7d
hsa-miR-1308_v15.0
hsa-miR-590-5p
hsa-miR-324-5p
hsa-miR-320a
hsa-miR-1260
hsa-miR-19a
hsa-miR-17
hsa-miR-1914*
hsa-miR-331-3p
hsa-miR-374b
hsa-miR-17*
hsa-miR-140-3p
hsa-miR-652
hsa-miR-125b
hsa-miR-29b
hsa-miR-181b
hsa-let-7e
hsa-miR-92a
hsa-miR-148b
hsa-miR-1974_v14.0
hsa-miR-15b
hsa-miR-342-3p
hsa-miR-20a
hsa-miR-16
hsa-miR-1288
hsa-miR-1280
hsa-miR-21
hsa-miR-106b
hsa-miR-29a
hsa-miR-19b
hsa-miR-93
hsa-miR-1975_v15.0
hsa-miR-99a
hsa-miR-30b
hsa-let-7i
hsa-miR-195
hsa-miR-126
hsa-miR-375
hsa-miR-22
hsa-miR-30d
hsa-miR-200c
hsa-miR-27a
hsa-miR-1202
hsa-miR-185
hsa-miR-340
hsa-miR-151-5p
hsa-miR-18a
hsa-miR-16-2*
hsa-miR-30a
hsa-miR-301a
hsa-let-7g
hsa-miR-24
hsa-miR-130b
hsa-miR-1977_v14.0
hsa-let-7a
hsa-miR-96
hsa-miR-27b
hsa-miR-429
hsa-miR-200b
hsa-miR-128
hsa-miR-575
hsa-miR-103a
hsa-let-7f
hsa-miR-425
hsa-miR-18b
hsa-miR-7
hsa-miR-1290
hsa-miR-374a
hsa-miR-1973
hsa-miR-30e
hsa-miR-513b
hsa-miR-378
hsa-miR-34b*
hsa-miR-29c
hsa-miR-892b
hsa-miR-15b*
hsa-miR-148a
hsa-miR-203
hsa-miR-15a
hsa-miR-101
hsa-miR-335
hsa-miR-365
hsa-miR-629
hsa-miR-98
hsa-miR-183*
hsa-miR-622
hsa-miR-106b*
hsa-miR-503
hsa-let-7a*
hsa-miR-615-3p
hsa-miR-200b*
hsa-miR-200c*
hsa-miR-940
hsa-miR-210
hsa-miR-19b-1*
hsa-miR-93*
hsa-miR-501-5p
hsa-miR-638
hsa-miR-150*
hsa-let-7d*
hsa-miR-1183
hsa-miR-1226*
hsa-miR-1238
hsa-miR-190b
hsa-miR-30d*
hsa-miR-33b*
hsa-miR-370
hsa-miR-586
hsa-miR-134
hsa-miR-1915
hsa-miR-181d
hsa-miR-1207-5p
hsa-miR-1268
hsa-miR-720
hsa-miR-7-1*
hsa-miR-140-5p
hsa-miR-181a*
hsa-miR-513a-5p
hsa-miR-1225-5p
hsa-miR-574-5p
hsa-miR-183
hsa-miR-1224-5p
hsa-miR-301b
hsa-miR-1275
hsa-miR-219-5p
hsa-miR-1274b_v16.0
hsa-miR-497
hsa-miR-361-5p
hsa-miR-125a-3p
hsa-miR-505
hsa-miR-193a-3p
hsa-miR-125a-5p
hsa-miR-939
hsa-miR-423-5p
IR 6 Gy
2 8 24 (h)
hsa-miR-107
hsa-miR-182
hsa-miR-26b
hsa-let-7c
hsa-miR-454
hsa-miR-23b
hsa-miR-20b
hsa-miR-424
hsa-miR-26a
hsa-miR-200a
hsa-miR-23a
hsa-miR-186
hsa-miR-30c
hsa-miR-494
hsa-miR-1305
hsa-miR-212
hsa-miR-100
hsa-miR-1979_v15.0
hsa-miR-487b
hsa-miR-335*
hsa-miR-324-3p
hsa-miR-32
hsa-miR-513c
hsa-miR-196b
hsa-miR-338-5p
hsa-miR-1826_v15.0
-6.5 5.5 (log2)
Supplementary Figure S1. Extended datasets of 229 IR-responsive miRNAs in MCF7 cells.

## Slide 2
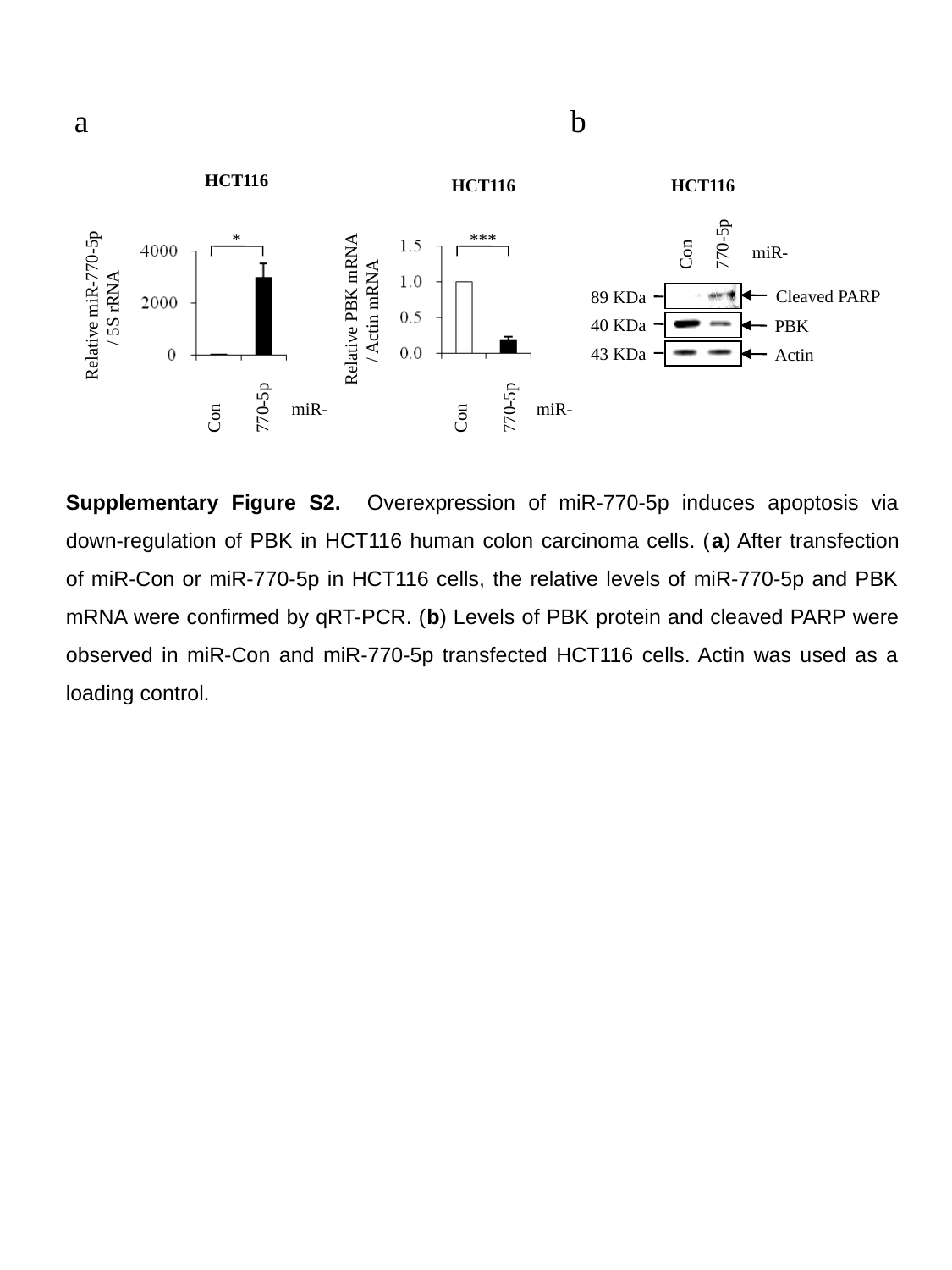

a
b
HCT116
HCT116
HCT116
770-5p
Con
Cleaved PARP
89 KDa
40 KDa
PBK
43 KDa
Actin
miR-
*
 Relative miR-770-5p
/ 5S rRNA
770-5p
Con
miR-
***
 Relative PBK mRNA
/ Actin mRNA
770-5p
miR-
Con
Supplementary Figure S2. Overexpression of miR-770-5p induces apoptosis via down-regulation of PBK in HCT116 human colon carcinoma cells. (a) After transfection of miR-Con or miR-770-5p in HCT116 cells, the relative levels of miR-770-5p and PBK mRNA were confirmed by qRT-PCR. (b) Levels of PBK protein and cleaved PARP were observed in miR-Con and miR-770-5p transfected HCT116 cells. Actin was used as a loading control.

## Slide 3
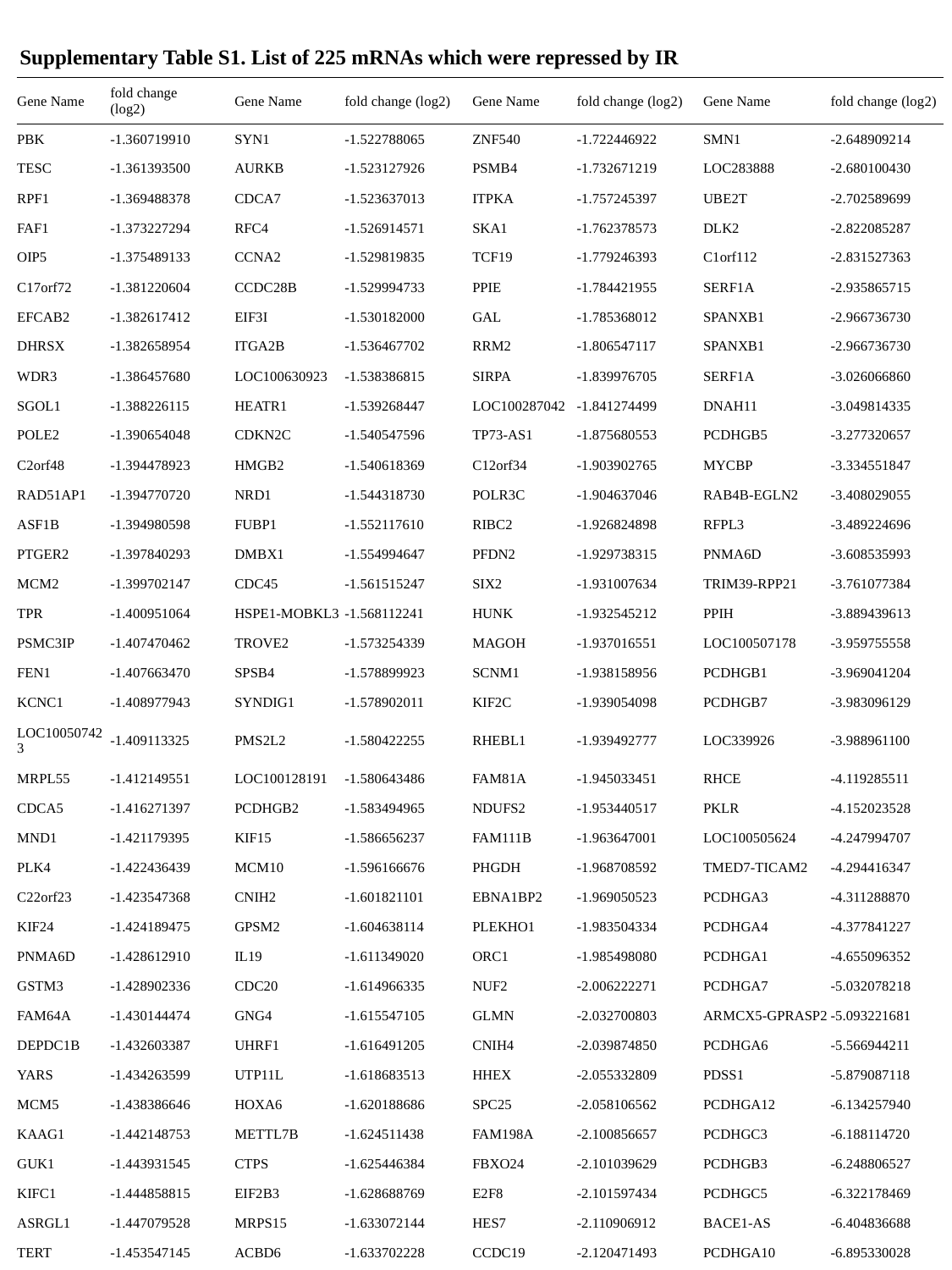

Supplementary Table S1. List of 225 mRNAs which were repressed by IR
| Gene Name | fold change (log2) | Gene Name | fold change (log2) | Gene Name | fold change (log2) | Gene Name | fold change (log2) |
| --- | --- | --- | --- | --- | --- | --- | --- |
| PBK | -1.360719910 | SYN1 | -1.522788065 | ZNF540 | -1.722446922 | SMN1 | -2.648909214 |
| TESC | -1.361393500 | AURKB | -1.523127926 | PSMB4 | -1.732671219 | LOC283888 | -2.680100430 |
| RPF1 | -1.369488378 | CDCA7 | -1.523637013 | ITPKA | -1.757245397 | UBE2T | -2.702589699 |
| FAF1 | -1.373227294 | RFC4 | -1.526914571 | SKA1 | -1.762378573 | DLK2 | -2.822085287 |
| OIP5 | -1.375489133 | CCNA2 | -1.529819835 | TCF19 | -1.779246393 | C1orf112 | -2.831527363 |
| C17orf72 | -1.381220604 | CCDC28B | -1.529994733 | PPIE | -1.784421955 | SERF1A | -2.935865715 |
| EFCAB2 | -1.382617412 | EIF3I | -1.530182000 | GAL | -1.785368012 | SPANXB1 | -2.966736730 |
| DHRSX | -1.382658954 | ITGA2B | -1.536467702 | RRM2 | -1.806547117 | SPANXB1 | -2.966736730 |
| WDR3 | -1.386457680 | LOC100630923 | -1.538386815 | SIRPA | -1.839976705 | SERF1A | -3.026066860 |
| SGOL1 | -1.388226115 | HEATR1 | -1.539268447 | LOC100287042 | -1.841274499 | DNAH11 | -3.049814335 |
| POLE2 | -1.390654048 | CDKN2C | -1.540547596 | TP73-AS1 | -1.875680553 | PCDHGB5 | -3.277320657 |
| C2orf48 | -1.394478923 | HMGB2 | -1.540618369 | C12orf34 | -1.903902765 | MYCBP | -3.334551847 |
| RAD51AP1 | -1.394770720 | NRD1 | -1.544318730 | POLR3C | -1.904637046 | RAB4B-EGLN2 | -3.408029055 |
| ASF1B | -1.394980598 | FUBP1 | -1.552117610 | RIBC2 | -1.926824898 | RFPL3 | -3.489224696 |
| PTGER2 | -1.397840293 | DMBX1 | -1.554994647 | PFDN2 | -1.929738315 | PNMA6D | -3.608535993 |
| MCM2 | -1.399702147 | CDC45 | -1.561515247 | SIX2 | -1.931007634 | TRIM39-RPP21 | -3.761077384 |
| TPR | -1.400951064 | HSPE1-MOBKL3 | -1.568112241 | HUNK | -1.932545212 | PPIH | -3.889439613 |
| PSMC3IP | -1.407470462 | TROVE2 | -1.573254339 | MAGOH | -1.937016551 | LOC100507178 | -3.959755558 |
| FEN1 | -1.407663470 | SPSB4 | -1.578899923 | SCNM1 | -1.938158956 | PCDHGB1 | -3.969041204 |
| KCNC1 | -1.408977943 | SYNDIG1 | -1.578902011 | KIF2C | -1.939054098 | PCDHGB7 | -3.983096129 |
| LOC100507423 | -1.409113325 | PMS2L2 | -1.580422255 | RHEBL1 | -1.939492777 | LOC339926 | -3.988961100 |
| MRPL55 | -1.412149551 | LOC100128191 | -1.580643486 | FAM81A | -1.945033451 | RHCE | -4.119285511 |
| CDCA5 | -1.416271397 | PCDHGB2 | -1.583494965 | NDUFS2 | -1.953440517 | PKLR | -4.152023528 |
| MND1 | -1.421179395 | KIF15 | -1.586656237 | FAM111B | -1.963647001 | LOC100505624 | -4.247994707 |
| PLK4 | -1.422436439 | MCM10 | -1.596166676 | PHGDH | -1.968708592 | TMED7-TICAM2 | -4.294416347 |
| C22orf23 | -1.423547368 | CNIH2 | -1.601821101 | EBNA1BP2 | -1.969050523 | PCDHGA3 | -4.311288870 |
| KIF24 | -1.424189475 | GPSM2 | -1.604638114 | PLEKHO1 | -1.983504334 | PCDHGA4 | -4.377841227 |
| PNMA6D | -1.428612910 | IL19 | -1.611349020 | ORC1 | -1.985498080 | PCDHGA1 | -4.655096352 |
| GSTM3 | -1.428902336 | CDC20 | -1.614966335 | NUF2 | -2.006222271 | PCDHGA7 | -5.032078218 |
| FAM64A | -1.430144474 | GNG4 | -1.615547105 | GLMN | -2.032700803 | ARMCX5-GPRASP2 | -5.093221681 |
| DEPDC1B | -1.432603387 | UHRF1 | -1.616491205 | CNIH4 | -2.039874850 | PCDHGA6 | -5.566944211 |
| YARS | -1.434263599 | UTP11L | -1.618683513 | HHEX | -2.055332809 | PDSS1 | -5.879087118 |
| MCM5 | -1.438386646 | HOXA6 | -1.620188686 | SPC25 | -2.058106562 | PCDHGA12 | -6.134257940 |
| KAAG1 | -1.442148753 | METTL7B | -1.624511438 | FAM198A | -2.100856657 | PCDHGC3 | -6.188114720 |
| GUK1 | -1.443931545 | CTPS | -1.625446384 | FBXO24 | -2.101039629 | PCDHGB3 | -6.248806527 |
| KIFC1 | -1.444858815 | EIF2B3 | -1.628688769 | E2F8 | -2.101597434 | PCDHGC5 | -6.322178469 |
| ASRGL1 | -1.447079528 | MRPS15 | -1.633072144 | HES7 | -2.110906912 | BACE1-AS | -6.404836688 |
| TERT | -1.453547145 | ACBD6 | -1.633702228 | CCDC19 | -2.120471493 | PCDHGA10 | -6.895330028 |
| APITD1 | -1.454693026 | RNF157 | -1.636478028 | GK3P | -2.128361864 | LY75-CD302 | -7.070915003 |
| PRIM1 | -1.456426293 | ZNF367 | -1.639893194 | HSPB11 | -2.136174221 | PCDHGB6 | -7.229321820 |
| SMYD2 | -1.458236165 | PHF19 | -1.642856718 | PSMA5 | -2.149676825 | PCDHGB4 | -8.194251530 |
| CDC25A | -1.459359984 | KRTCAP2 | -1.644714332 | LOC100129250 | -2.193303846 | CBWD3 | -8.799717728 |
| ESPL1 | -1.465861984 | NRTN | -1.654852371 | SGTB | -2.217744630 | PTCD1 | -14.446515880 |
| LOC254559 | -1.468182197 | FBN1 | -1.661363524 | NASP | -2.235866104 | ZNF816-ZNF321P | -14.760849180 |
| NRM | -1.470472423 | LOC401097 | -1.663789458 | ITGB3BP | -2.249673699 | H3F3A | -15.992659480 |
| UGT1A9 | -1.476292545 | RCN3 | -1.664375567 | PRIMA1 | -2.263176950 | NBL1 | -16.039752090 |
| CLSPN | -1.482444859 | ASPM | -1.668722202 | NT5C1B-RDH14 | -2.306051531 | URGCP-MRPS24 | -17.009545480 |
| KRT5 | -1.483361851 | MKI67 | -1.672235628 | EXO1 | -2.320121441 | FIGF | -18.460858210 |
| ERCC6L | -1.493691052 | TBXA2R | -1.673082100 | ACTL8 | -2.344435494 | LOC285819 | -25.845205370 |
| CDCA8 | -1.497723228 | WDR76 | -1.678930058 | RAD54L | -2.378757200 | TMEFF1 | -29.063181980 |
| SPC24 | -1.502197003 | DNAH14 | -1.685847503 | LOC100130557 | -2.400404889 | BAGE4 | -31.724501640 |
| TXNRD3NB | -1.503561943 | ILF2 | -1.697055771 | LOC100507463 | -2.425639743 | CORO7-PAM16 | -72.539048730 |
| FZD9 | -1.507469185 | CDCA3 | -1.706907207 | B3GAT2 | -2.444728813 | PRR5-ARHGAP8 | -84.648102510 |
| CNTNAP2 | -1.515888279 | LOXL3 | -1.713260314 | TBCE | -2.468619026 | TMX2-CTNND1 | -161.765142800 |
| PKMYT1 | -1.516004507 | PLGLB1 | -1.717034016 | KLHL23 | -2.511405381 | | |
| SCN4A | -1.517496366 | GK | -1.719719986 | ZNF670 | -2.536757384 | | |
| SKA3 | -1.518714167 | AKAP2 | -1.720500393 | GPR89B | -2.578671417 | | |

## Slide 4
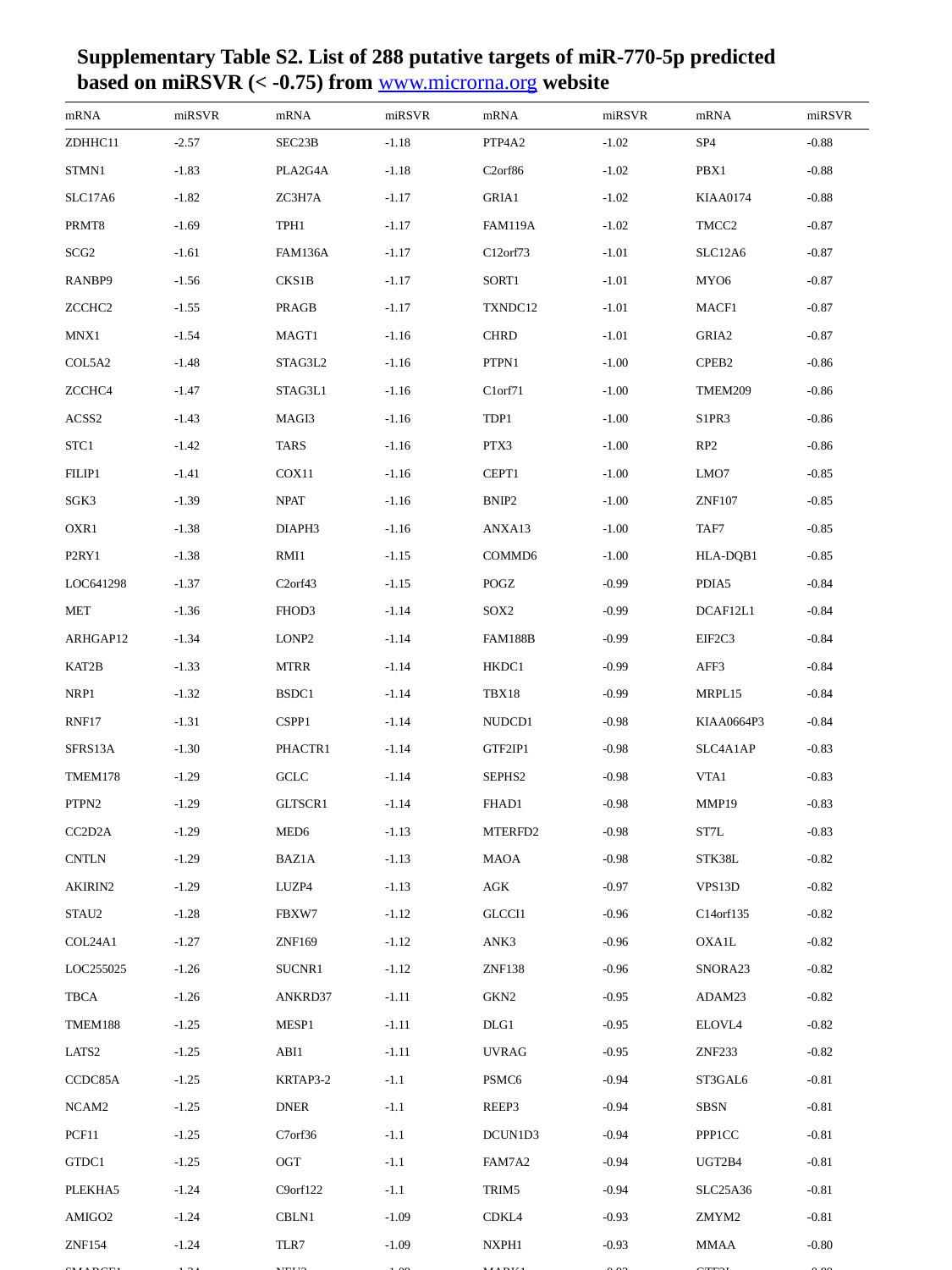

Supplementary Table S2. List of 288 putative targets of miR-770-5p predicted based on miRSVR (< -0.75) from www.microrna.org website
| mRNA | miRSVR | mRNA | miRSVR | mRNA | miRSVR | mRNA | miRSVR |
| --- | --- | --- | --- | --- | --- | --- | --- |
| ZDHHC11 | -2.57 | SEC23B | -1.18 | PTP4A2 | -1.02 | SP4 | -0.88 |
| STMN1 | -1.83 | PLA2G4A | -1.18 | C2orf86 | -1.02 | PBX1 | -0.88 |
| SLC17A6 | -1.82 | ZC3H7A | -1.17 | GRIA1 | -1.02 | KIAA0174 | -0.88 |
| PRMT8 | -1.69 | TPH1 | -1.17 | FAM119A | -1.02 | TMCC2 | -0.87 |
| SCG2 | -1.61 | FAM136A | -1.17 | C12orf73 | -1.01 | SLC12A6 | -0.87 |
| RANBP9 | -1.56 | CKS1B | -1.17 | SORT1 | -1.01 | MYO6 | -0.87 |
| ZCCHC2 | -1.55 | PRAGB | -1.17 | TXNDC12 | -1.01 | MACF1 | -0.87 |
| MNX1 | -1.54 | MAGT1 | -1.16 | CHRD | -1.01 | GRIA2 | -0.87 |
| COL5A2 | -1.48 | STAG3L2 | -1.16 | PTPN1 | -1.00 | CPEB2 | -0.86 |
| ZCCHC4 | -1.47 | STAG3L1 | -1.16 | C1orf71 | -1.00 | TMEM209 | -0.86 |
| ACSS2 | -1.43 | MAGI3 | -1.16 | TDP1 | -1.00 | S1PR3 | -0.86 |
| STC1 | -1.42 | TARS | -1.16 | PTX3 | -1.00 | RP2 | -0.86 |
| FILIP1 | -1.41 | COX11 | -1.16 | CEPT1 | -1.00 | LMO7 | -0.85 |
| SGK3 | -1.39 | NPAT | -1.16 | BNIP2 | -1.00 | ZNF107 | -0.85 |
| OXR1 | -1.38 | DIAPH3 | -1.16 | ANXA13 | -1.00 | TAF7 | -0.85 |
| P2RY1 | -1.38 | RMI1 | -1.15 | COMMD6 | -1.00 | HLA-DQB1 | -0.85 |
| LOC641298 | -1.37 | C2orf43 | -1.15 | POGZ | -0.99 | PDIA5 | -0.84 |
| MET | -1.36 | FHOD3 | -1.14 | SOX2 | -0.99 | DCAF12L1 | -0.84 |
| ARHGAP12 | -1.34 | LONP2 | -1.14 | FAM188B | -0.99 | EIF2C3 | -0.84 |
| KAT2B | -1.33 | MTRR | -1.14 | HKDC1 | -0.99 | AFF3 | -0.84 |
| NRP1 | -1.32 | BSDC1 | -1.14 | TBX18 | -0.99 | MRPL15 | -0.84 |
| RNF17 | -1.31 | CSPP1 | -1.14 | NUDCD1 | -0.98 | KIAA0664P3 | -0.84 |
| SFRS13A | -1.30 | PHACTR1 | -1.14 | GTF2IP1 | -0.98 | SLC4A1AP | -0.83 |
| TMEM178 | -1.29 | GCLC | -1.14 | SEPHS2 | -0.98 | VTA1 | -0.83 |
| PTPN2 | -1.29 | GLTSCR1 | -1.14 | FHAD1 | -0.98 | MMP19 | -0.83 |
| CC2D2A | -1.29 | MED6 | -1.13 | MTERFD2 | -0.98 | ST7L | -0.83 |
| CNTLN | -1.29 | BAZ1A | -1.13 | MAOA | -0.98 | STK38L | -0.82 |
| AKIRIN2 | -1.29 | LUZP4 | -1.13 | AGK | -0.97 | VPS13D | -0.82 |
| STAU2 | -1.28 | FBXW7 | -1.12 | GLCCI1 | -0.96 | C14orf135 | -0.82 |
| COL24A1 | -1.27 | ZNF169 | -1.12 | ANK3 | -0.96 | OXA1L | -0.82 |
| LOC255025 | -1.26 | SUCNR1 | -1.12 | ZNF138 | -0.96 | SNORA23 | -0.82 |
| TBCA | -1.26 | ANKRD37 | -1.11 | GKN2 | -0.95 | ADAM23 | -0.82 |
| TMEM188 | -1.25 | MESP1 | -1.11 | DLG1 | -0.95 | ELOVL4 | -0.82 |
| LATS2 | -1.25 | ABI1 | -1.11 | UVRAG | -0.95 | ZNF233 | -0.82 |
| CCDC85A | -1.25 | KRTAP3-2 | -1.1 | PSMC6 | -0.94 | ST3GAL6 | -0.81 |
| NCAM2 | -1.25 | DNER | -1.1 | REEP3 | -0.94 | SBSN | -0.81 |
| PCF11 | -1.25 | C7orf36 | -1.1 | DCUN1D3 | -0.94 | PPP1CC | -0.81 |
| GTDC1 | -1.25 | OGT | -1.1 | FAM7A2 | -0.94 | UGT2B4 | -0.81 |
| PLEKHA5 | -1.24 | C9orf122 | -1.1 | TRIM5 | -0.94 | SLC25A36 | -0.81 |
| AMIGO2 | -1.24 | CBLN1 | -1.09 | CDKL4 | -0.93 | ZMYM2 | -0.81 |
| ZNF154 | -1.24 | TLR7 | -1.09 | NXPH1 | -0.93 | MMAA | -0.80 |
| SMARCE1 | -1.24 | NEU3 | -1.09 | MARK1 | -0.93 | GTF2I | -0.80 |
| C6orf134 | -1.24 | ADAM22 | -1.09 | MFI2 | -0.92 | ANKAR | -0.80 |
| CDC123 | -1.24 | IGF2BP2 | -1.07 | C12orf42 | -0.92 | MBNL3 | -0.80 |
| STAG3L3 | -1.24 | FMO1 | -1.07 | KIAA1024 | -0.92 | PKD2 | -0.79 |
| DCIM1D1 | -1.24 | RBM46 | -1.07 | FAM7A1 | -0.92 | SH3RF2 | -0.79 |
| MATN2 | -1.24 | VCAM1 | -1.07 | PACRGL | -0.92 | TMEM135 | -0.79 |
| TP53BP1 | -1.23 | GTPBP4 | -1.07 | CCDC67 | -0.92 | AFG3L1 | -0.79 |
| C1orf150 | -1.23 | BTG1 | -1.07 | DNAJA2 | -0.92 | ASCC1 | -0.79 |
| HDGFRP3 | -1.23 | SACS | -1.07 | RAPGEF4 | -0.92 | ST3GAL3 | -0.79 |
| HERC3 | -1.22 | ZNF331 | -1.06 | CARD8 | -0.92 | FGD2 | -0.79 |
| MAP3K1 | -1.22 | VWA3B | -1.06 | WDR7 | -0.91 | BCR | -0.79 |
| SLC30A5 | -1.22 | PLXDC2 | -1.06 | RAD23B | -0.91 | NUP35 | -0.79 |
| CSGALNACT1A | -1.22 | CCDC102B | -1.05 | TMEM222 | -0.91 | PBK | -0.78 |
| LOC440040 | -1.22 | RNF2 | -1.05 | RYR1 | -0.91 | EPHA6 | -0.78 |
| GRP98 | -1.21 | MRC1 | -1.05 | FBXO33 | -0.91 | ATF7IP | -0.78 |
| MAPKAP1 | -1.21 | DNAJC24 | -1.05 | ZNF514 | -0.91 | PDIK1L | -0.78 |
| SAMD3 | -1.21 | CARNS1 | -1.04 | KCNJ3 | -0.91 | UBQLN1 | -0.78 |
| C9orf3 | -1.21 | RAP1GDS1 | -1.04 | GLRX3 | -0.90 | SVEP1 | -0.77 |
| CCDC60 | -1.20 | HPGD | -1.04 | CAPN2 | -0.90 | ZRANB3 | -0.77 |
| EIF4G3 | -1.20 | SLC25A24 | -1.04 | FAT4 | -0.90 | ESRRB | -0.77 |
| FAM108C1 | -1.19 | PECI | -1.03 | GMFB | -0.90 | TACSTD2 | -0.76 |
| MYST1 | -1.19 | ABHD1 | -1.03 | BMP6 | -0.90 | WNT10B | -0.76 |
| AGTR2 | -1.19 | CCDC150 | -1.03 | KCNJ1 | -0.89 | NLRC3 | -0.76 |
| MESDC1 | -1.19 | FAM86D | -1.03 | SUV420H1 | -0.89 | TM2D2 | -0.76 |
| CISD2 | -1.19 | CNR1 | -1.03 | FAM7A3 | -0.89 | C20orf72 | -0.75 |
| HERC4 | -1.18 | NAA38 | -1.03 | MTPN | -0.89 | C13orf34 | -0.75 |
| KCNMB2 | -1.18 | KCNG3 | -1.03 | SAMD7 | -0.89 | LRFN5 | -0.75 |
| XRN2 | -1.18 | ASXL1 | -1.03 | CDKAL1 | -0.89 | ARMCX2 | -0.75 |
| FAH2 | -1.18 | LPIN1 | -1.03 | FLRT3 | -0.89 | ZNF606 | -0.75 |
| NAP1L1 | -1.18 | XPNPEP3 | -1.02 | THAP4 | -0.89 | TUBA4B | -0.75 |
| UBR3 | -1.18 | KCNK1 | -1.02 | NRSN1 | -0.88 | HIRA | -0.75 |
